# Progression-guided spatiotemporal memory transformers for accurate and consistent longitudinal brain tumor segmentation

**DOI:** 10.1038/s41598-026-53337-2

**Published:** 2026-05-25

**Authors:** Sandeep Kumar Mathivanan, Shamala K. Subramaniam, Dafik, Sunder R, Sangeetha S.K.B, Siva Shankar S

**Affiliations:** 1 School of Computing Science and Engineering, Galgotias University, Greater Noida, Uttar Pradesh 203201 India; 2 Department of Communication Technology and Network, Faculty of Computer Science and Information Technology, , Universiti Putra Malaysia (UPM), Serdang, Selangor 43400 Malaysia; 3 Department of Mathematics, Faculty of Mathematics and Natural Sciences, , University of Jember, Jember, Indonesia; 4 Manipal Institute of Technology Bengaluru, Manipal Academy of Higher Education, Manipal, India; 5 Department of Computer Science and Engineering, KG Reddy College of Engineering and Technology, Hyderabad, Telangana 501504 India

**Keywords:** Longitudinal brain tumor segmentation, Spatiotemporal memory, Progression-aware temporal modeling, Boundary-enhanced transformer, MRI, Tumor evolution dynamics, Cross-time structural alignment, Dice score, Temporal consistency, Deep learning, Cancer, Computational biology and bioinformatics, Mathematics and computing, Oncology

## Abstract

For monitoring the progression of the disease and the efficacy of treatment, it is essential to segment the brain tumor. The majority of the available deep learning models in use today are based on discrete time points without considering the continuity of the process, which causes irregular shapes of the tumors in the subsequent images. In this study, a model called Progression-Guided Spatiotemporal Memory Transformer (PGSMT) has been proposed, which has a unique design to overcome the constraints. A progression-aware temporal memory module, where the latent tumor representation is built up across successive MRI scans, a cross-time structural alignment mechanism, where the consistency of tumor morphology is preserved with the ability to accommodate pathological changes, and a boundary-enhanced transformer encoder, where the spatial dependencies are captured and stored for precise boundary delineation, are the three major components of the proposed framework. PGSMT learns the temporal weighting, making it possible for the network to distinguish between noise and actual progression, unlike other temporal fusion methods. When compared with the Convolutional Neural Network (CNN), hybrid CNN-Transformer, and Transformer models, the proposed PGSMT outperforms these models in the BraTS longitudinal benchmark dataset. PGSMT shows statistically significant improvements (*p* < 0.05), with 88.1% Dice for the enhancing tumor, 90.2% Dice for the tumor core, and 93.0% Dice for the total tumor. while reducing inter-scan volumetric inconsistencies significantly. The relevant therapeutic interest in the dynamics of changing tumor volumes is substantiated by attention analysis. With respect to the empirical evaluation on the BraTS longitudinal data set, the results achieve 88.1% Dice for the enhancing tumor, 90.2% Dice for the tumor core, and 93.0% Dice for the total tumor, demonstrating statistically significant improvements (*p* < 0.05) compared to traditional methods, while the results indicate increased stability in terms of temporal variance.

## Introduction

The effective clinical management, diagnosis, treatment planning, and tracking of the progression of the disease are highly dependent on the accurate and effective segmentation of the brain tumor in the MRI scan. The high spatial heterogeneity and rapid temporal growth rates of brain tumors, especially gliomas, are major issues for both computational models and radiologists. The accuracy in the estimation of the volume and characterization of the tumor morphology is inconsistent, considering the tedious and highly variable nature of the manual segmentation of the tumor region in the MRI scans. Manual segmentation is tedious, time-consuming, and subject to inter-observer variability^1,2^. The problem becomes more complicated in the longitudinal study, considering the accurate tracking of the progression, regression, or response of the tumor to the treatment for the evaluation of the effectiveness of the treatment and the planning of the treatment strategy for individual patients. The development of automated segmentation methods with the ability to accurately depict tumor boundaries and changes over time has become an important area of research in the field of neuro-oncology.

Segmentation of brain tumors has been significantly enhanced in the last ten years with the advent of deep learning approaches. To accurately segment the sub-regions of the tumor, such as the enhancing tumor, tumor core, and the entire tumor, Convolutional Neural Networks (CNNs), especially the architecture of the U-Net and its extensions, have been successful in extracting hierarchical spatial features from the MRI scans. To capture the contextual information and simulate the dependencies between the slices, hybrid models, especially those integrating CNNs with Recurrent Neural Networks (RNNs) and attention mechanisms, have been explored^1,3,4^. Through the employment of these self-attention mechanisms, the transformer-based models have even enhanced their ability to recognize long-range spatial dependencies, thereby improving the management of irregular tumor shapes and boundary delineation. The major drawback of these models is that they process individual MRI scans at specific times.

The reliability of volumetric and morphological evaluations, especially in longitudinal studies, is restricted by the temporal continuity of the employed method, resulting in irregular tumor segmentations during the study. Tumor development and response evaluation involve the comprehension of temporal changes. Tumor development is heterogeneous because of the presence of certain microenvironmental factors and the impact of therapeutic interventions. Tumor development varies at different locations. Failure to account for temporal relationships between consecutive scans lead to incorrect assessments of therapy effectiveness, false changes in expected tumor volumes, and misrepresentation of progression patterns. Segmentation discrepancies may be compounded by registration errors, image noise, and time-point acquisition variation, which is a major challenge for longitudinal analysis.

Models that can accommodate temporal information while maintaining spatial accuracy and robustness against imaging errors are necessary for solving these issues. Simple temporal fusion approaches, such as stacking images of several time points or using temporal recurrent layers, have been explored; however, their clinical potential is low since they often do not adequately differentiate noise from biological changes. At the same time, the number of brain cancer types demands precise detection of the tumor’s sub-regional areas, which are of particular importance in treatment. A key factor in the assessment of the efficacy of treatment, particularly following radiation and chemotherapy, is the enhancing tumor area, which signifies the growth of the tumor. The assessment of the tumor’s aggression and potential invasion of the surrounding brain tissue can be made from the tumor’s core, which contains necrotic and non-enhancing areas.

The entire tumor area is relevant in the context of surgical resections and potential neurological issues. The segmentation of the tumor’s areas is challenging in the context of changes in intensity, morphology, and geographic extent over time. Although conventional deep learning frameworks are not equipped with special tools to ensure temporal consistency or tumor characteristics’ changes, conventional intensity-based or classical machine learning approaches often fail to cope with the changes. The necessity to develop segmentation techniques that incorporate temporal information has been emphasized with the availability of several longitudinal brain tumor datasets, including the BraTS longitudinal benchmark. These datasets contain various scans of the tumor at different time intervals, showing the organic growth of the tumor as well as the effect of treatment.

For the evaluation of the longitudinal consistency and segmentation accuracy of the tumor, volumetric consistency, temporal variance, and dice similarity coefficient are important. The most advanced CNN model, CNN-Transformer hybrid model, and the Transformer model exhibit high segmentation accuracy at a given time point but are associated with considerable variations with follow-up scans. The practical use of these models is also limited by considerable variations. The accuracy and reliability of automated segmentation are compromised by the fact that the models currently lack interpretable mechanisms to ascertain the areas that impact the models’ predictions with time. Longitudinal brain tumor segmentation is challenged not only by technical challenges but also those that impact patient care. Tumor growth rate estimation errors, inappropriate modification of treatment plans, and less than optimal patient outcomes can arise from inaccurate or suboptimal tumor segmentation.

The proper evaluation of treatment, the monitoring of the remaining or new tumor areas, and the early detection of tumor progression can be assisted by the accurate automation of the process of segmenting the images in successive scans. The provision of standardized measurements of the volumes of the tumors can improve the reliability of clinical trials, the identification of biomarkers of prognosis, and radiomic research. There is a growing consensus that models of segmentation need to use techniques that reflect the growth of the tumors while maintaining localization accuracy, which goes beyond the static approach of a single-time-point analysis. Recent research has shown the feasibility and potential benefits of the inclusion of time information in the process of segmenting brain tumors, despite the challenges. Techniques such as memory modules, temporal attention processes, and recurrent feature aggregation have demonstrated potential for detecting patterns of progression over time.

The techniques highlight the significance of finding a balance between spatial accuracy and temporal continuity, enabling adaptive weighing of information from previous scans as well as sensitivity to actual changes in diseases. Long-range connections in tumor tissues can be modeled using complex attention-based structures, enabling improved morphological accuracy as well as precise demarcation of boundaries. Such advancements demonstrate that it is feasible to design a system that ensures not only excellent accuracy in segmenting images but also continuity over time, thus addressing a major gap in neuro-oncology. The need for a reliable and consistent brain tumor segmentation in longitudinal images acquired by MRI has been the driving force behind this research. The challenges of tumor formation, diversity in terms of structure, and noise in the longitudinal images are still not met by the traditional deep learning methods, despite their performance in static images.

The assessment of the efficacy of the treatment, timely decision-making in the treatment process, and adaptation of the treatment plan to the patient require the monitoring of the growth of the tumor. This study has the potential to bridge the gap between the clinical need and the high-performing algorithms. Thus, an aim of the study is to develop techniques that can capture both the temporal and spatial characteristics of tumors, as well as being interpretable.

The longitudinal segmentation of tumors in the brain becomes complex compared to that at a single time point since the tumor changes in size, shape, and appearance with each successive scan. Tumor progression, treatment response, and errors within the imaging process like noise and registration issues are what make these differences happen. Traditional methods generate inconsistent segmentation results along the different time points due to their independent processing of each image. This could have negative impacts on medical decisions and give inaccurate interpretations of the growth of the tumor.

The main contributions are

- To design a spatiotemporal segmentation framework (PGSMT) that accurately segments tumor sub-regions (ET, TC, WT) using multimodal MRI data, evaluated using Dice Similarity Coefficient (DSC) and Hausdorff Distance.
- To ensure temporal consistency across longitudinal scans by incorporating progression-guided memory and temporal attention mechanisms, evaluated using temporal variance and volumetric consistency metrics.
- To improve robustness in challenging scenarios such as small tumor regions and irregular progression patterns, validated through qualitative visual analysis.

## Related study

### Longitudinal brain tumor segmentation methods

Longitudinal medical image analysis plays a critical role in monitoring disease progression, evaluating treatment response, and supporting clinical decision-making. Early deep learning approaches for brain tumor analysis predominantly relied on Convolutional Neural Networks (CNNs) operating on single time-point MRI scans. While these models achieved strong spatial segmentation accuracy, they failed to capture temporal dependencies across follow-up scans, leading to inconsistent tumor delineation and unreliable progression assessment.

To address this limitation, spatiotemporal learning frameworks integrating CNNs with recurrent architectures such as ConvLSTM were introduced to model inter-scan dependencies and tumor evolution patterns^5,9,1^. Similarly, spatial-temporal fusion techniques aggregate information across multiple time points to improve segmentation consistency^7^. Despite these advancements, such approaches often suffer from limited scalability, sensitivity to temporal noise, and insufficient modeling of long-range temporal relationships, resulting in unstable longitudinal predictions.

### Temporal memory and recurrence-based approaches

Recent developments have explored transformer-based architectures for modeling temporal dependencies due to their ability to capture global contextual relationships. Spatiotemporal transformers have been applied to brain tumor classification and grading tasks by jointly modeling spatial appearance and temporal evolution^6^. Time-aware transformer variants and time-distance vision transformers further incorporate temporal intervals between scans to improve longitudinal modeling^9,18^.

Alternative sequence modeling approaches, such as Mamba-based architectures, have demonstrated computational efficiency in longitudinal CT lesion segmentation tasks^17^. However, these models are primarily designed for general sequence learning and lack explicit mechanisms to distinguish true biological progression from imaging noise, which is critical for reliable tumor progression modeling. Furthermore, many transformer-based longitudinal models focus on classification or prediction rather than voxel-wise dense segmentation with temporal consistency constraints^20,22^.

### Deformable and alignment-based consistency methods

Maintaining structural consistency across longitudinal scans is essential for accurate tumor tracking. Deformable and shape-based modeling approaches, including transformer-based morphological analysis frameworks, have been proposed to capture progressive anatomical changes over time^8,12,11^. These methods emphasize global shape evolution and structural correspondence across scans.

However, existing approaches typically operate at a coarse representation level and do not incorporate feature-level deformable alignment within segmentation frameworks. As a result, they are unable to effectively handle local anatomical variations, subtle tumor shifts, and registration inconsistencies, leading to residual structural misalignment and segmentation errors across time points.

### Boundary-aware transformer-based segmentation

Transformer-based segmentation models, including 3D transformer-U-Net hybrids and multi-scale attention frameworks, have significantly improved spatial accuracy and boundary delineation in brain tumor segmentation tasks^3,14^. These methods leverage global attention mechanisms and hierarchical feature representations to capture complex tumor structures.

Despite their strong spatial performance, these models are inherently designed for single-time-point segmentation and do not enforce temporal consistency across longitudinal scans. Consequently, boundary predictions may vary significantly between successive scans due to minor anatomical changes, noise, or misregistration. Additionally, conventional CNN-based and transfer learning approaches^15^ operate under static assumptions and fail to address longitudinal variability in tumor morphology.

### Limitations of existing approaches and positioning of the proposed method

Despite substantial progress, existing methods do not simultaneously address the three fundamental requirements of longitudinal brain tumor segmentation: temporal consistency, structural alignment, and boundary-aware refinement.

Several advanced deep learning models that facilitate better feature representation and segmentation have been developed recently in brain tumor segmentation research. To model finer spatial properties, the EffUNet + + and DCRUNet + + adopt advanced U-net architectures, featuring residual and depth-wise convolutions^17^. To model contextual relationships in MRI images, the modified recurrent residual attention U-Net utilizes attention and recurrence learning^18^. Deep feature extraction approaches advance representation learning, which is important for both segmentation and classification tasks^19^. More robust spatiotemporal frameworks are needed since, even though there have been many advances in the field, most models focus on single-time point analysis and do not tackle longitudinal consistency or temporal evolution issues explicitly^21^. demonstrate that pseudo-label refinement and symmetric regularization significantly improve segmentation accuracy in semi-supervised medical imaging tasks. Adaptive strategies like iterative pseudo-labeling with copy-paste supervision enhance model generalization by effectively utilizing unlabeled data and improving robustness.

Recurrent and transformer-based temporal models capture sequential dependencies but lack mechanisms to differentiate true tumor progression from imaging noise, resulting in temporal instability and over-smoothing of tumor evolution. Deformable alignment methods focus on global structural correspondence but do not integrate learnable feature-level alignment into segmentation pipelines, leading to persistent inconsistencies across time points. Meanwhile, transformer-based segmentation frameworks achieve high spatial accuracy but operate under single-time-point assumptions, failing to enforce cross-time boundary coherence^13,16^.

To overcome these limitations, the proposed Progression-Guided Spatiotemporal Memory Transformer (PGSMT) introduces a unified framework consisting of three key components:

- Progression-aware temporal memory (PATM): captures tumor evolution dynamics by adaptively modeling temporal dependencies and suppressing noise-driven fluctuations.
- Cross-time structural alignment module (CTSAM): performs deformable feature-level alignment to preserve morphological continuity across scans.
- Boundary-enhanced transformer encoder (BETE): integrates boundary-aware attention to ensure precise and temporally stable tumor delineation.

By jointly addressing temporal reliability, structural consistency, and boundary precision, the proposed approach provides a comprehensive solution for voxel-wise longitudinal brain tumor segmentation, which is not achieved by existing methods.

## System methodology

### Dataset description

Two publicly available datasets were used in the development of the proposed PGSMT framework, namely, the BraTS 2020 Glioma Segmentation Dataset for independent external validation and the BraTS Longitudinal Benchmark Dataset (https://www.kaggle.com/datasets/talhaumar/brats2020-correct-dataset-training-validation, https://www.kaggle.com/datasets/awsaf49/brats2020-training-data, https://www.synapse.org/#!Synapse:syn28740011/wiki/70861) for primary training and testing.

The BraTS Longitudinal dataset comprises 612 volumetric MRI scans from 164 glioma patients, each undergoing multiple follow-up sessions. For each patient, three to five longitudinal MRI scans are available, enabling modeling of tumor progression over time. Each scan includes four modalities: FLAIR, T1, T1ce, and T2, with a standardized spatial resolution of 240 × 240 × 155 voxels at 1 mm³ isotropic resolution, stored in 3D NIfTI format. Accordingly, each time point is represented as a 4-channel volumetric tensor of size 4 × 240 × 240 × 155.

The ground truth annotations follow the standard BraTS labeling protocol, where each voxel is assigned one of four mutually exclusive classes: background (0), necrotic/non-enhancing tumor core (1), peritumoral edema (2), and enhancing tumor (4). During training, the model directly predicts these multi-class voxel-wise labels. For evaluation, clinically relevant composite regions are derived as follows: Enhancing Tumor (ET) corresponds to label 4, Tumor Core (TC) includes labels {1, 4}, and Whole Tumor (WT) includes labels {1, 2, 4}. These regions are overlapping and are used solely for evaluation following the BraTS protocol.

The dataset exhibits substantial variability in tumor characteristics, ranging from small, localized lesions (< 5 cm^3^) to large infiltrative tumors (> 120 cm^3^). Both Low-Grade Glioma (LGG) and High-Grade Glioma (HGG) cases are included, collected from multiple institutions, ensuring diversity in anatomical structures and intensity distributions.

For experimental consistency, the longitudinal dataset was partitioned at the patient level to preserve temporal continuity. Specifically:

- 115 patients (≈ 428 volumes) were used for training.
- 16 patients (≈ 60 volumes) were used for validation (hyperparameter tuning and model selection).
- 33 patients (≈ 124 volumes) were reserved for internal testing.

All time points belonging to a given patient were assigned to the same subset, and no subjects were excluded.

The BraTS 2020 dataset, consisting of single time-point multi-modal MRI scans, was used exclusively for external testing to evaluate cross-dataset generalization. A subset of 50 cases was selected based on consistent modality structure and spatial resolution. This dataset was not used for training, validation, or hyperparameter tuning, ensuring an unbiased evaluation protocol under different acquisition conditions. All hyperparameters were fixed prior to testing and were not tuned on the external validation dataset to avoid bias.

Prior to the actual training of the models, all the MRI volumes in the BraTS 2020 dataset and the BraTS Longitudinal Benchmark Dataset underwent the same preprocessing steps, ensuring numerical stability, spatial consistency, and intensity harmonization. To remove low-frequency intensity inhomogeneity artifacts, the N4 bias field correction method was used for all the modalities. The image I_corr_ is produced by dividing the observed image I_obs_ by the estimated bias field B(x), and the corrected image I_corr_, given the position x, is I_corr_ (x) = I_obs_ (x)/B(x). To ensure the spatial correlation, all the modalities were skull-stripped and firmly aligned to the same anatomical template space. Using z-score normalization in the non-zero brain region, intensity normalization was carried out separately for every modality.

For each modality volume *\:X*, normalized intensities were computed as $\:{X}_{norm}=(X-{\mu\:}_{X})/{\sigma\:}_{X}$, where $\:{\mu\:}_{X}\:$and $\:{\sigma\:}_{X}\:$denote the mean and standard deviation of non-zero voxels in that volume. This normalization also harmonizes the intensity distributions across different institutions, which reduces inter-scanner variability. For all volumes, a spatial dimension of 240 × 240 × 155 was used, and resampling was done with isotropic resolution (1 mm³) ^3^o ensure spatial consistency across subjects and time points. The input tensor of each time point was represented as X^t^ ∈ R^(4 × 240 × 240 × 155)^, where four channels indicate FLAIR, T1, T1ce, and T2 modalities. The ground truth annotations follow the standard BraTS labeling scheme with four mutually exclusive classes: background (0), necrotic/non-enhancing tumor core (1), peritumoral edema (2), and enhancing tumor (4). The model is trained using these multi-class voxel-wise labels. For evaluation, three clinically relevant regions are derived:

- ET: label 4.
- TC: labels {1, 4}.
- WT: labels {1, 2, 4}.

These regions are overlapping and are used only for evaluation, not as independent training targets.

The whole tumor mask is defined as $\:WT=\{v\mid\:y(v)\in\:\{\mathrm{1,2},4\left\}\right\}$,where $\:y\left(v\right)$denotes the voxel label. Stable transformer-based spatiotemporal learning is made possible by this preprocessing pipeline, which guarantees statistically normalized feature distributions, uniform spatial alignment, and modality consistency across longitudinal and external validation datasets.

### Problem formulation

The objective of longitudinal tumor segmentation is to accurately segment the tumor sub-regions from consecutive Magnetic Resonance Imaging (MRI) scans conducted at various periods from the same patient. Let the set X = {X_1_,X_2_,…,X_T_} be the definition of a longitudinal sequence. Let X_t_ be a multi-modal MRI scan from the set X, where X_t_ ∈ R^(C×H×W)^. C is the number of imaging modes, and H, W are the resolution dimensions. Let the set Y = {Y_1_,Y_2_,…,Y_T_} be the set of related ground truth masks, where Y_T_ denotes the voxel-wise segmentation map with mutually exclusive multi-class labels defined by the BraTS protocol. The model performs multi-class segmentation, while ET, TC, and WT are derived during evaluation. For the longitudinal brain tumor segmentation, the inter-scan dependencies must be captured to address the tumor growth, regression, and treatment-related structural changes. This contrasts with the conventional segmentation method, where the set Y_t_ = f_θ_(X_t_) is estimated independently at every time point. The clinical reliability of the conventional method is compromised by independent modeling, which causes irregular volume predictions and border changes in the consecutive scans.

To solve this limitation, the problem of segmentation is recast as a conditional spatiotemporal learning problem, which follows the form Y_t_ = f_θ_ (X_t_|H_(t−1)_), where H_(t−1)_ represents the latent space for the evolution of the tumor, and the prediction at the current time point depends on the current scan and the cumulative history. The learning goal, as specified by the loss function, maximizes the accuracy of the predictions with respect to the entire sequence while maintaining sensitivity to the actual changes in the disease and the physiological growth patterns of the tumor. The problem of segmentation, transitions from being a collection of individual predictions in the spatial domain to the spatiotemporal domain.

### Longitudinal sequence construction and temporal modeling

The MRI scans taken longitudinally were organized into time series for each patient based on the chronological order of acquisition. To achieve chronological consistency, the MRI scans were sorted based on their index in each patient’s records since the data contains consecutive follow-up scans but lacks any timestamp information. The number of time points per patient varies between three and five. The length of the time series was kept at T = 5 to make sure that each can be effectively trained using batch processing. Zero padding was applied to preserve the temporality of the series without including any artificial alterations for patients who did not have as many scans. This involved replicating the last available scan. As all-time series fall within the given limit, there is no need for truncation.

Batching in sequence-wise fashion was done for the training process, whereby every batch contains entire sequences belonging to different patients. Parallel processing can be applied while maintaining the dependencies within the sequence. The temporal memory aware of the progression is defined at t = 1 as

$${{\rm{M}}_0} = 0$$

In this case, the network operates as a spatial segmentation network with no constraints of temporal continuity or alignment. Cross-time structural alignment and temporal memory updates are incorporated incrementally from later time points (t > 1). This method does not make use of the actual time gaps between scans; rather, it uses only scans to predict the temporal progression. This makes the methodology more robust for datasets in which the time gap information is not necessarily available. The methodology does assume is that the time relationship between the scans is relatively linear. The limitation of this particular methodology is the assumption of evenly spaced time gaps, which likely affect the modeling of the tumor growth rate.

### Overall architecture of PGSMT

Compared with ConvLSTM-based models, static–dynamic transformers, and existing memory-based segmentation approaches, the proposed PGSMT is fundamentally distinguished by: (1) progression-aware temporal memory for modeling true tumor evolution, (2) cross-time structural alignment for maintaining spatial consistency, and (3) boundary-enhanced transformer encoding for precise delineation. This unified design enables simultaneous optimization of temporal continuity, spatial coherence, and boundary accuracy, which is not achieved by existing methods.

**Fig. 1:**
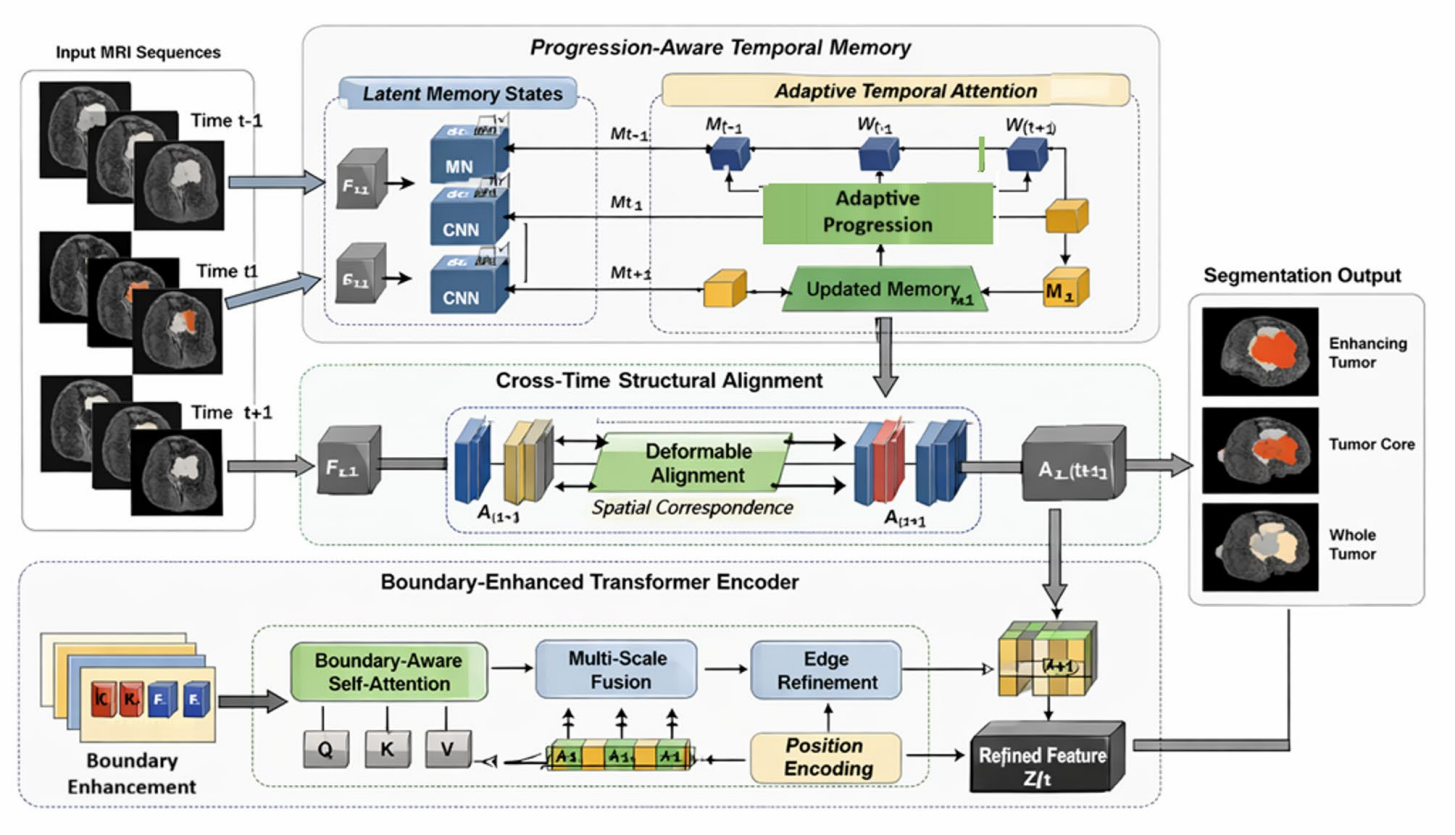
Proposed PGSMT framework.

The proposed Progression-Guided Spatiotemporal Memory Transformer (PGSMT) as shown in Fig. 1 follows a structured spatiotemporal pipeline for longitudinal brain tumor segmentation. Given sequential multi-modal MRI scans $\:\mathcal{X}=\{{X}_{1},{X}_{2},\dots\:,{X}_{T}\}$, spatial representations are first extracted using a feature encoder $\:{F}_{t}=\phi\:\left({X}_{t}\right)$, where $\:{F}_{t}\in\:{\mathbb{R}}^{D\times\:{H}^{{\prime\:}}\times\:{W}^{{\prime\:}}}$. These features are then processed by a progression-aware temporal memory module that integrates historical tumor evolution information. The prediction at time *\:t*is conditioned on prior context as $\:{\widehat{Y}}_{t}={f}_{\theta\:}({F}_{t}\mid\:{M}_{t-1})$, where $\:{M}_{t-1}$denotes the latent memory state. The memory is updated using a progression residual $\:{{\Delta\:}}_{t}={F}_{t}-{F}_{t-1}$to explicitly encode tumor evolution, yielding $\:{M}_{t}=\alpha\:{M}_{t-1}+(1-\alpha\:)\left({\Psi\:}\right({F}_{t},{M}_{t-1})+{W}_{{\Delta\:}}{{\Delta\:}}_{t})$, where $\:\alpha\:$is a learnable retention coefficient and $\:{\Psi\:}(\cdot\:)$represents adaptive temporal aggregation.

The temporally enriched features are subsequently refined through cross-time structural alignment to preserve morphological continuity. A deformable warping operator $\:\mathcal{W}(\cdot\:)$aligns prior features as $\:{\widehat{F}}_{t-1}=\mathcal{W}\left({F}_{t-1}\right)$, and structural consistency is enforced via $\:{\mathcal{L}}_{align}=\parallel\:{F}_{t}-{\widehat{F}}_{t-1}{\parallel\:}_{2}^{2}$. The aligned representations are then processed by a boundary-enhanced transformer encoder employing modified self-attention ${\rm{Attentio}}{{\rm{n}}_b}(Q,K,V) = {\rm{Softmax}}\left( {\frac{{Q{K^{\rm T}} + \lambda \:B}}{{\sqrt d }}} \right)V$, where *\:B*encodes boundary priors. Finally, voxel-wise predictions are generated through a decoder $\:{\widehat{Y}}_{t}=\mathrm{Softmax}\left(Conv\right({Z}_{t}\left)\right)$, Softmax produces voxel-wise probabilities over the four mutually exclusive classes, optimized using a composite objective $\:{\mathcal{L}}_{total}={\mathcal{L}}_{Dice}+{\lambda\:}_{1}{\mathcal{L}}_{align}+{\lambda\:}_{2}{\mathcal{L}}_{temp}$, where $\:{\mathcal{L}}_{temp}=\parallel\:{\widehat{Y}}_{t}-\mathcal{W}\left({\widehat{Y}}_{t-1}\right){\parallel\:}_{1}$enforces longitudinal consistency.

#### Spatial feature encoding

For each time point *\:t*, the input multi-modal MRI scan $\:{X}_{t}$is first processed through a convolutional neural network backbone to extract hierarchical spatial representations, formulated as $\:{F}_{t}=\phi\:\left({X}_{t}\right)$, where $\:{F}_{t}\in\:{\mathbb{R}}^{D\times\:{H}^{{\prime\:}}\times\:{W}^{{\prime\:}}}$. Here, H′ and W′ denote the spatial resolution after down sampling, while D denotes the dimension of feature embeddings. The encoder captures essential information regarding anatomical features, multi-scale contextual information, and tumor appearance features, which are vital for precise sub-region segmentation. The aggregation of local features is efficiently facilitated by convolutional operations, which also enable spatial coherence, a vital aspect of later operations, namely, alignment and attention. The computational cost of this operation, based on convolutional kernel operations over feature channels, is approximately O(H′W′D^2^). The feature maps F_t_, which are generated in later stages of the PGSMT model, are highly discriminative and concise representations.

#### Progression-aware temporal memory module (PATM)

##### Algorithm 1

Progression-guided spatiotemporal memory update.

Input: Spatial feature tensor at time *\:t*, $\:{F}_{t}\in\:{\mathbb{R}}^{D\times\:{H}^{{\prime\:}}\times\:{W}^{{\prime\:}}}$; previous feature tensor $\:{F}_{t-1}$; previous latent memory state $\:{M}_{t-1}\in\:{\mathbb{R}}^{L\times\:D}$.

Output: Updated latent memory state $\:{M}_{t}\in\:{\mathbb{R}}^{L\times\:D}$.

Here, *\:D*denotes embedding dimensionality, $\:{H}^{{\prime\:}},{W}^{{\prime\:}}$represent spatial resolution after encoding, $\:N={H}^{{\prime\:}}{W}^{{\prime\:}}$is the number of spatial tokens, *\:L*is the number of memory slots, and $\:{H}_{a}$denotes the number of attention heads with $\:{d}_{h}=D/{H}_{a}$.

Step 1: The spatial feature tensor $\:{F}_{t}$is reshaped into a sequence of token embeddings as

1 $$\:{\mathrm{F}}_{t}=\mathrm{vec}\left({F}_{t}\right)\in\:{\mathbb{R}}^{N\times\:D},$$

where the operator $\:\mathrm{vec}(\cdot\:)$flattens the spatial dimensions into *\:N*tokens, and each token $\:{\mathrm{F}}_{t}^{\left(i\right)}\in\:{\mathbb{R}}^{D}$represents the embedding of the $\:{i}^{th}$spatial location.

Step 2: The token embeddings are normalized using layer normalization,

2 $$\:{\stackrel{\prime }{\mathrm{F}}}_{t}=\mathrm{LN}\left({\mathrm{F}}_{t}\right),$$

where LN standardizes each token along the feature dimension *\:D*, ensuring numerical stability and consistent feature distribution across time steps.

Step 3: The normalized tokens are projected into multiple query subspaces using learnable matrices,

3 $$\:{\mathrm{Q}}_{t}^{\left(h\right)}={\stackrel{\prime }{\mathrm{F}}}_{t}{W}_{q}^{\left(h\right)},{W}_{q}^{\left(h\right)}\in\:{\mathbb{R}}^{D\times\:{d}_{h}},$$

where $\:h=1,\dots\:,{H}_{a}$, and $\:{d}_{h}$is the dimensionality of each attention head, generating query representations for temporal matching.

Step 4: The previous memory state $\:{M}_{t-1}$is linearly transformed into the key embedding space,

4 $$\:{\mathrm{K}}_{t-1}^{\left(h\right)}={M}_{t-1}{W}_{k}^{\left(h\right)},{W}_{k}^{\left(h\right)}\in\:{\mathbb{R}}^{D\times\:{d}_{h}},$$

where $\:{W}_{k}^{\left(h\right)}$is a learnable key projection matrix and $\:{\mathrm{K}}_{t-1}^{\left(h\right)}\in\:{\mathbb{R}}^{L\times\:{d}_{h}}$encodes historical memory slots into comparable subspaces.

Step 5: The same previous memory is projected into the value embedding space as

5 $$\:{\mathrm{V}}_{t-1}^{\left(h\right)}={M}_{t-1}{W}_{v}^{\left(h\right)},{W}_{v}^{\left(h\right)}\in\:{\mathbb{R}}^{D\times\:{d}_{h}},$$

where $\:{W}_{v}^{\left(h\right)}$is a learnable value projection matrix and the resulting values store transferable historical context.

Step 6: The scaled similarity matrix between current queries and historical keys is computed as

6 $$\:{{\Lambda\:}}_{t}^{\left(h\right)}=\frac{{\mathrm{Q}}_{t}^{\left(h\right)}({\mathrm{K}}_{t-1}^{\left(h\right)}{)}^{\mathrm{T}}}{\sqrt{{d}_{h}}},$$

where $\:{{\Lambda\:}}_{t}^{\left(h\right)}\in\:{\mathbb{R}}^{N\times\:L}$measures correlation strength and the factor $\:\sqrt{{d}_{h}}$stabilizes high-dimensional dot products.

Step 7: The similarity matrix is normalized into attention weights through softmax activation,

7 $$\:{\mathrm{A}}_{t}^{\left(h\right)}=\mathrm{Softmax}\left({{\Lambda\:}}_{t}^{\left(h\right)}\right)+\varepsilon\mathrm{I},$$

where $\:\varepsilon$is a small positive constant for numerical robustness and $\:\mathrm{I}$ denotes the identity matrix.

Step 8: The temporally refined representations are obtained by aggregating values according to attention weights,

8 $$\:{\stackrel{\sim}{\mathrm{M}}}_{t}^{\left(h\right)}={\mathrm{A}}_{t}^{\left(h\right)}{\mathrm{V}}_{t-1}^{\left(h\right)},$$

where each token integrates weighted historical information proportional to its temporal relevance.

Step 9: The outputs from all attention heads are concatenated and linearly fused as

9 $$\:{\stackrel{\sim}{\mathrm{M}}}_{t}=\mathrm{Concat}({\stackrel{\sim}{\mathrm{M}}}_{t}^{\left(1\right)},\dots\:,{\stackrel{\sim}{\mathrm{M}}}_{t}^{\left({H}_{a}\right)}){W}_{o},$$

where $\:{W}_{o}\in\:{\mathbb{R}}^{D\times\:D}$is a learnable output projection matrix ensuring unified embedding dimensionality.

Step 10: The inter-scan tumor evolution residual is computed as

10 $$\:{{\Delta\:}}_{t}={\stackrel{\prime }{\mathrm{F}}}_{t}-{\stackrel{\prime }{\mathrm{F}}}_{t-1},$$

where $\:{{\Delta\:}}_{t}\in\:{\mathbb{R}}^{N\times\:D}$captures temporal changes in embedding space reflecting tumor growth or regression.

Step 11: The residual is transformed into a nonlinear progression embedding using

11 $$\:{\mathrm{E}}_{t}={\phi\:}_{g}({{\Delta\:}}_{t}{W}_{{\Delta\:}}+{b}_{{\Delta\:}}),$$

where $\:{W}_{{\Delta\:}}\in\:{\mathbb{R}}^{D\times\:D}$is a learnable transformation matrix, $\:{b}_{{\Delta\:}}$is bias, and $\:{\phi\:}_{g}(\cdot\:)$denotes GELU activation.

Step 12: The global magnitude of progression is estimated through

12 $$\:{\gamma\:}_{t}=\sigma,\left(\frac{1}{N}\sum\:_{i=1}^{N}\parallel\:{{\Delta\:}}_{t}^{\left(i\right)}{\parallel\:}_{2}^{2}{W}_{\gamma\:}\right),$$

where $\:\parallel\:\cdot\:{\parallel\:}_{2}$denotes Euclidean norm, $\:{W}_{\gamma\:}$is a learnable scalar weight, and $\:\sigma\:(\cdot\:)$ is the sigmoid function constraining $\:{\gamma\:}_{t}\in\:\left(\mathrm{0,1}\right)$.

Step 13: The nonlinear progression embedding is adaptively gated as

13 $$\:{\widehat{\mathrm{E}}}_{t}={\gamma\:}_{t}\odot\:{\mathrm{E}}_{t},$$

where $\:\odot\:$represents element-wise multiplication, ensuring that stronger biological changes exert greater influence.

Step 14: An adaptive retention coefficient is computed as

14 $$\:{\alpha\:}_{t}=\sigma\:({W}_{\alpha\:}{\mu\:}_{t}+{b}_{\alpha\:}),{\mu\:}_{t}=\frac{1}{N}\sum\:_{i=1}^{N}{\stackrel{\prime }{\mathrm{F}}}_{t}^{\left(i\right)},$$

where $\:{W}_{\alpha\:}$and $\:{b}_{\alpha\:}$are learnable parameters and $\:{\alpha\:}_{t}\in\:\left(\mathrm{0,1}\right)$regulates memory preservation.

Step 15: The final memory state is updated according to

15 $$\:{M}_{t}={\alpha\:}_{t}{M}_{t-1}+(1-{\alpha\:}_{t})\left({\stackrel{\sim}{\mathrm{M}}}_{t}+{\widehat{\mathrm{E}}}_{t}\right),$$

where the convex combination ensures balanced integration of historical stability and progression-aware refinement within the latent spatiotemporal memory manifold.

The interaction between the latent memory slots and the spatial token embeddings mainly affects the computational complexity of the progression-guided temporal attention mechanism. In the scaled dot-product attention, the computation of the correlations between N_queries_ and L_keys_ in the embedding dimension D contributes a complexity of O(LDH′W′), where N = H′W′ spatial tokens and L memory slots. This term arises from the matrix multiplication ${\mathrm{Q}}_{T}{\mathrm{K}}_{t-1}^{{\rm T}}\in\:{\mathbb{R}}^{N\times\:L}$and subsequent weighted aggregation with values. In contrast, the memory update operation, which involves gated residual fusion and linear transformations within the $\:L\times\:D$latent memory space, incurs a comparatively lower complexity of $\:\mathcal{O}\left(LD\right)$. Since $\:L\ll\:{H}^{{\prime\:}}{W}^{{\prime\:}}$in practical settings, the overall design remains computationally scalable and efficient for moderate memory sizes while preserving rich temporal modeling capacity.

##### Algorithm 2

Cross-time structural alignment module (CTSAM).

Input: Current spatial feature tensor $\:{F}_{t}\in\:{\mathbb{R}}^{D\times\:{H}^{{\prime\:}}\times\:{W}^{{\prime\:}}}$; previous spatial feature tensor $\:{F}_{t-1}\in\:{\mathbb{R}}^{D\times\:{H}^{{\prime\:}}\times\:{W}^{{\prime\:}}}$.

Output: Aligned previous feature map $\:{\widehat{F}}_{t-1}$and structural alignment loss $\:{\mathcal{L}}_{align}$.

Here, *\:D*denotes embedding dimensionality, $\:{H}^{{\prime\:}},{W}^{{\prime\:}}$represent spatial resolution, and $\:N={H}^{{\prime\:}}{W}^{{\prime\:}}$denotes total spatial locations. The operator $\:\odot\:$represents element-wise multiplication, and $\:\sigma\:(\cdot\:)$ denotes the sigmoid activation function.

Step 1: The current feature tensor $\:{F}_{t}$is first normalized using channel-wise normalization to stabilize feature magnitudes, producing

16 $$\:{\stackrel{\prime }{F}}_{t}=\mathrm{Norm}\left({F}_{t}\right),$$

where normalization ensures consistent structural comparison across time points.

Step 2: A deformable offset estimation network $\:\psi\:(\cdot\:)$, parameterized by learnable weights $\:{\theta\:}_{\psi\:}$, predicts a dense spatial displacement field

17 $$\:{O}_{t}=\psi\:\left({\stackrel{\prime }{F}}_{t}\right),{O}_{t}\in\:{\mathbb{R}}^{2\times\:{H}^{{\prime\:}}\times\:{W}^{{\prime\:}}},$$

where each spatial location contains horizontal and vertical displacement components.

Step 3: The offset tensor $\:{O}_{t}$is reshaped into coordinate-wise displacement vectors

18 $$\:{O}_{t}(i,j)=({\Delta\:}{x}_{i,j},{\Delta\:}{y}_{i,j}),$$

where $\:\left(i,j\right)$ indexes spatial position and $\:{\Delta\:}x,{\Delta\:}y$ denote learned spatial shifts.

Step 4: A sampling grid $\:{G}_{t}\in\:{\mathbb{R}}^{{H}^{{\prime\:}}\times\:{W}^{{\prime\:}}\times\:2}$ is constructed by adding offsets to the regular coordinate grid

19 $$\:{G}_{t}(i,j)=(i+{\Delta\:}{x}_{i,j},{\hspace{0.25em}\hspace{0.05em}}j+{\Delta\:}{y}_{i,j}),$$

where the grid defines new sampling coordinates for alignment.

Step 5: The previous feature tensor $\:{F}_{t-1}$is warped using a differentiable bilinear interpolation operator

20 $$\:{\widehat{F}}_{t-1}=\mathcal{W}({F}_{t-1},{O}_{t}),$$

where $\:\mathcal{W}(\cdot\:)$denotes bilinear resampling over grid $\:{G}_{t}$, producing aligned features $\:{\widehat{F}}_{t-1}\in\:{\mathbb{R}}^{D\times\:{H}^{{\prime\:}}\times\:{W}^{{\prime\:}}}$.

Step 6: Bilinear interpolation computes each aligned feature as

21 $$\:{\widehat{F}}_{t-1}(i,j)=\sum\:_{m,n}{F}_{t-1}(m,n)\cdot\:\mathrm{m}\mathrm{a}\mathrm{x}(\mathrm{0,1}-\mid\:{i}^{{\prime\:}}-m\mid\:)\cdot\:\mathrm{m}\mathrm{a}\mathrm{x}(\mathrm{0,1}-\mid\:{j}^{{\prime\:}}-n\mid\:),$$

where $\:\left({i}^{{\prime\:}},{j}^{{\prime\:}})={G}_{t}(i,j\right)$and summation occurs over neighboring pixels.

Step 7: The structural discrepancy between current and aligned features is computed as

22 $$\:{R}_{t}={F}_{t}-{\widehat{F}}_{t-1},$$

where $\:{R}_{t}\in\:{\mathbb{R}}^{D\times\:{H}^{{\prime\:}}\times\:{W}^{{\prime\:}}}$captures residual morphological differences.

Step 8: A temporal difference map is additionally computed as

23 $$\:{D}_{t}=\mid\:{F}_{t}-{F}_{t-1}\mid\:,$$

where absolute difference emphasizes magnitude of inter-scan variation.

Step 9: A structural reliability weight map is generated using

24 $$\:{W}_{t}=\sigma\:\left({D}_{t}\right),$$

where $\:{W}_{t}\in\:(\mathrm{0,1}{)}^{D\times\:{H}^{{\prime\:}}\times\:{W}^{{\prime\:}}}$adaptively suppresses regions undergoing strong biological evolution.

Step 10: The weighted residual is computed via element-wise modulation

25 $$\:{\stackrel{\sim}{R}}_{t}={W}_{t}\odot\:{R}_{t},$$

where $\:\odot\:$ensures evolving regions contribute less to alignment penalty.

Step 11: The structural consistency loss is defined as

26 $$\:{\mathcal{L}}_{align}=\parallel\:{\stackrel{\sim}{R}}_{t}{\parallel\:}_{2}^{2},$$

where $\:\parallel\:\cdot\:{\parallel\:}_{2}$denotes Frobenius norm across spatial and channel dimensions.

Step 12: Expanding the loss explicitly,

27 $$\:{{\cal L}_{align}} = \sum {\:_{d = 1}^D} \sum {\:_{i = 1}^{{H^{\prime {\kern 1pt} }}}} \sum {\:_{j = 1}^{{W^{\prime {\kern 1pt} }}}} {\left( {W_t^{\left( {d,i,j} \right)} \cdot \left( {F_t^{\left( {d,i,j} \right)} - \hat F_{t - 1}^{\left( {d,i,j} \right)}} \right)} \right)^2},$$

which enforces structural continuity while respecting adaptive weighting.

Step 13: A smoothness regularizer is optionally applied to the offset field

28 $$\:{\mathcal{L}}_{smooth}=\sum\:_{i,j}\parallel\:\nabla\:{O}_{t}(i,j){\parallel\:}_{2}^{2},$$

where $\:\nabla\:$denotes spatial gradient, promoting physically plausible deformations.

Step 14: The total alignment objective is formulated as

29 $$\:{\mathcal{L}}_{CTSAM}={\mathcal{L}}_{align}+{\lambda\:}_{s}{\mathcal{L}}_{smooth},$$

where $\:{\lambda\:}_{s}$ controls deformation smoothness strength.

Step 15: The aligned feature tensor $\:{\widehat{F}}_{t-1}$ is forwarded to subsequent temporal modeling modules, ensuring morphologically consistent yet biologically adaptive longitudinal representation.

This algorithm enables deformable structural alignment across time while preventing over-constraining dynamically evolving tumor regions, thereby preserving both anatomical continuity and pathological progression fidelity.

##### Algorithm 3

Boundary-enhanced transformer encoding and segmentation optimization.

Input: Spatial feature tensor $\:{F}_{t}\in\:{\mathbb{R}}^{D\times\:{H}^{{\prime\:}}\times\:{W}^{{\prime\:}}}$; aligned previous segmentation $\:{S}_{t-1}$.

Output: Predicted segmentation map $\:{\widehat{Y}}_{t}$ and total optimization loss $\:{\mathcal{L}}_{total}$.

*\:D*denotes embedding dimension, $\:{H}^{{\prime\:}},{W}^{{\prime\:}}$represent spatial resolution, $\:N={H}^{{\prime\:}}{W}^{{\prime\:}}$, *\:S*is number of transformer scales, $\:\lambda\:,{\lambda\:}_{1},{\lambda\:}_{2}$ are balancing hyperparameters, and $\:\nabla\:$denotes spatial gradient operator.

Boundary-Enhanced Transformer Encoder (BETE).

Step 1: The spatial feature tensor $\:{F}_{t}$is reshaped into token embeddings

30 $$\:{\mathrm{F}}_{t}=\mathrm{vec}\left({F}_{t}\right)\in\:{\mathbb{R}}^{N\times\:D},$$

where each token corresponds to a spatial location in the encoded MRI feature map.

Step 2: A boundary prior representation is extracted using spatial gradient operators

31 $$\:{E}_{t}=\nabla\:{F}_{t},$$

where $\:\nabla\:$computes horizontal and vertical derivatives, producing edge-sensitive responses highlighting tumor margins.

Step 3: The gradient magnitude map is computed as

32 $$\:{G}_{t}(i,j)=\sqrt{\left({\partial\:}_{x}{F}_{t}{)}^{2}+({\partial\:}_{y}{F}_{t}{)}^{2}\right.},$$

where $\:\left(i,j\right)$denotes spatial indices and the magnitude emphasizes high-frequency boundary regions.

Step 4: The boundary affinity matrix is constructed by measuring pairwise similarity between edge tokens

33 $$\:{B}_{t}={\mathrm{E}}_{t}{\mathrm{E}}_{t}^{\mathrm{T\:}},$$

where $\:{\mathrm{E}}_{t}\in\:{\mathbb{R}}^{N\times\:D}$is the vectorized boundary embedding and $\:{B}_{t}\in\:{\mathbb{R}}^{N\times\:N}$encodes edge correlation strength.

Step 5: Query, key, and value projections are computed as

34 $$\:Q={\mathrm{F}}_{t}{W}_{q},K={\mathrm{F}}_{t}{W}_{k},V={\mathrm{F}}_{t}{W}_{v},$$

where $\:{W}_{q},{W}_{k},{W}_{v}\in\:{\mathbb{R}}^{D\times\:D}$are learnable projection matrices.

Step 6: The boundary-enhanced attention logits are computed as

35 $$\:{{\Omega\:}}_{t}=\frac{Q{K}^{\mathrm{T\:}}+\lambda\:{B}_{t}}{\sqrt{D}},$$

where $\:\lambda\:$controls the contribution of boundary affinity and $\:\sqrt{D}$ stabilizes scaling.

Step 7: Attention weights are obtained through softmax normalization

36 $$\:{A}_{b}=\mathrm{Softmax}\left({{\Omega\:}}_{t}\right),$$

where normalization ensures probabilistic weighting across spatial tokens.

Step 8: The boundary-aware contextual representation is computed as

37 $$\:{Z}_{t}^{\left(1\right)}={A}_{b}V,$$

where edge-relevant regions receive amplified contextual influence.

Step 9: The feature tensor $\:{F}_{t}$is processed at multiple scales using hierarchical transformer blocks

38 $$\:{T}_{s}\left({F}_{t}\right),s=1,\dots\:,S,$$

where each $\:{T}_{s}(\cdot\:)$operates at a distinct spatial resolution.

Step 10: Each scale produces a contextual embedding

39 $$\:{Z}_{t}^{\left(s\right)}={T}_{s}\left({F}_{t}\right),$$

where coarse scales capture global context and fine scales preserve boundary precision.

Step 11: The multi-scale outputs are fused through learnable weights

40 $$\:{Z}_{t}=\sum\:_{s=1}^{S}{w}_{s}{Z}_{t}^{\left(s\right)},$$

where $\:{w}_{s}$are normalized fusion coefficients satisfying $\:{\sum\:}_{s}{w}_{s}=1$.

Step 12: The fused representation is reshaped back into spatial tensor form and passed through convolutional decoding layers

41 $$\:{\widehat{Y}}_{t}=\mathrm{Softmax}\left(\mathrm{Conv}\right({Z}_{t}\left)\right),$$

where convolution aggregates local details and softmax produces class probabilities.

Step 13: The Dice loss for segmentation accuracy is computed as

42 $$\:{\mathcal{L}}_{Dice}=1-\frac{2\sum\:_{i}{\widehat{Y}}_{t}^{\left(i\right)}{Y}_{t}^{\left(i\right)}}{\sum\:_{i}{\widehat{Y}}_{t}^{\left(i\right)}+\sum\:_{i}{Y}_{t}^{\left(i\right)}},$$

where $\:{Y}_{t}$ denotes ground-truth segmentation.

Step 14: The segmentation task is formulated as a multi-class voxel-wise prediction problem. The segmentation loss is defined as:

43 $$\:{L}_{\mathrm{seg}}={L}_{\mathrm{Dice}}+{L}_{\mathrm{CE}}$$

The total loss is:

44 $$\:{L}_{\mathrm{total}}={L}_{\mathrm{seg}}+{\lambda\:}_{1}{L}_{\mathrm{align}}+{\lambda\:}_{2}{L}_{\mathrm{temp}}+{\lambda\:}_{s}{L}_{\mathrm{smooth}}$$

where $\:{L}_{\mathrm{align}}$: structural alignment loss, $\:{L}_{\mathrm{temp}}$: temporal consistency loss, $\:{L}_{\mathrm{smooth}}$: deformation smoothness loss.

Hyperparameters are set as $\:{\lambda\:}_{1}=0.3,{\lambda\:}_{2}=0.2,{\lambda\:}_{s}=0.1$. All losses are computed for each time point and summed across the longitudinal sequence. The weighting parameters λ₁ and λ₂ were selected via empirical tuning on the validation set using a coarse grid search.

A lightweight 3D convolutional module with three convolutional layers (kernel size 3 × 3 × 3) and ReLU activations is used to create the deformable offset network ψ(·). For aligning features across temporal frames, it produces dense spatial offset fields. Through end-to-end joint training of the deformable offset network and the main segmentation network, the alignment parameters can be optimized by backpropagating the total loss function. A very small number of extra parameters are introduced by the deformable offset network, which adds very little to the total complexity of the model. For ψ(·), no independent optimization or independent pre-training is carried out.

To ensure linear scaling of the temporal attention operations in relation to the spatial tokens N = H′W′ and the embedding dimension D, the size of the latent memory L_s_ is intentionally restricted to a narrow range, typically L = 8–16. The global self-attention mechanism inherently makes the complexity of the transformer backbone quadratic, O(N^2^). To maintain this term computationally tractable in practice, the depth of the encoder is carefully restricted and only used at lower spatial resolutions. Unlike the attention blocks, the progressive guidance memory update imposes negligible overhead in the form of lightweight matrix multiplications in the L x D latent space. As a result, even with the deliberate inclusion of the temporal modeling and structural alignment mechanisms, the computational complexity of the PGSMT model is found to be only marginally higher than that of contemporary CNN-Transformer architectures by a mere 8–12%. This formulation ensures full consistency between training targets, network output, and evaluation metrics, avoiding ambiguity between multi-class and multi-label segmentation settings.

##  Experimental results and discussion

### Experimental setup

A high-performance computing workstation equipped with two NVIDIA RTX 4090 graphics processing units, each having 24 GB of memory in the form of GDDR6X, an Intel Xeon Gold processor with 32 cores and a clock speed of 2.6 GHz, and a memory of 256 GB DDR4 was used for conducting all experiments. Distributed data parallelism was used for the training of the model to effectively handle complete 3D volumetric inputs. Ubuntu 22.04 LTS was used as the underlying operating system. The implementation was done in PyTorch, version 2.1, with CUDA and cuDNN, both version 8.9, accelerated in Python, version 3.10. NVIDIA Apex library was used to enable the model to support mixed precision, i.e., FP16, to speed up convergence and save memory. MONAI and NiBabel libraries were used for data preprocessing and augmentation to improve the overall performance of the model. For improving the overall performance of the model, spatial transformations, intensity standardization, elastic deformation, gamma correction, and random affine transformations were used.

The model was trained using a distributed data parallel setup with a batch size of 2 per GPU (effective batch size = 4). Due to memory constraints of full 3D volumes, training was performed using random 3D patches of size 128 × 128 × 128. Due to GPU memory limitations with full 3D volumes (240 × 240 × 155), patch-based training was adopted. The network was trained for 300 epochs with an initial learning rate of 1 × 10^−4^, using the AdamW optimizer with a weight decay of 1 × 10^−5^. A cosine annealing schedule was applied to progressively reduce the learning rate from 1 × 10^−4^ to a minimum of 1 × 10^−6^ over the training period. Early stopping was employed with a patience of 30 epochs, based on validation Dice stability across longitudinal sequences. For data augmentation, the following probabilistic transformations were applied:

- Random flipping (*p* = 0.5).
- Random rotation (*p* = 0.3).
- Elastic deformation (*p* = 0.2).
- Gamma intensity augmentation (*p* = 0.3).
- Random affine transformation (*p* = 0.2).

During inference, a sliding window strategy with 50% overlap was used to reconstruct full-resolution predictions. All experiments were conducted using 3 independent runs with different random seeds, and the reported results correspond to the mean performance across runs.

For the model optimization, gradient clipping with the maximum norm set at 1.0, cosine annealing learning rate scheduling, and AdamW with decoupled weight decay were used. Based on the evaluation of the dice stability for the longitudinal scans, early halting was applied. For the evaluation, clinically validated segmentation and longitudinal consistency measures were used. The Dice Similarity Coefficient (DSC) for the Enhancing Tumor (ET), Tumor Core (TC), and Whole Tumor (WT) was used for the evaluation of the accuracy in terms of space. The 95th percentile Hausdorff Distance (HD95) was used for the evaluation of the precision at the boundaries, and the reliability of the contours was determined. To evaluate the calculation of the tumor burden, Volumetric Similarity (VS) was used. Temporal Variance (TV) and Inter-Scan Volume Fluctuation (ISVF) were also calculated to assess the prediction stability across the follow-up scans to evaluate the longitudinal robustness of the predictions. Paired t-tests with 95% confidence intervals were also performed for the patient-wise holdout test set to ensure the results were statistically significant.

To extensively benchmark the proposed PGSMT, it was compared with eight models that use different architectural designs: (1) nnU-Net (powerful CNN with automated configuration); (2) 3D U-Net with deep supervision; (3) Attention U-Net with spatial attention gates; (4) Swin-UNet (hierarchical vision transformer); (5) UNETR (pure transformer encoder with CNN decoder); (6) hybrid CNN-transformer architecture combining CNN stem with global self-attention; (7) longitudinal segmentation model based on ConvLSTM to capture temporal recurrence; and (8) Static-Dynamic Coordinated Transformer designed for tumor growth modeling. To ensure the fairness of the comparative evaluation, all the baseline models were re-trained with the same pre-processing configuration and hyperparameter tuning strategies. To evaluate the scalability and deployability of the proposed PGSMT model, profiles of computational complexity, parameter numbers, GPU memory consumption, and inference delay were created.

### Quantitative segmentation performance

The standard deviation values are provided in Table 1 to highlight the consistency of the model with the longitudinal sequences and patients. The lowest variance is achieved by PGSMT across all tumors sub-regions while also having the highest mean Dice scores. For the Enhancing Tumor (ET), the 88.1 ± 2.1% is achieved by the PGSMT, while the 86.9 ± 2.6% is achieved by the Hybrid CNN-Transformer. The lower standard deviation indicates improved robustness to inter-patient differences and various progression patterns. Due to the small size of the lesions and the effect of treatment on the intensity values, the segmentation results for the Enhancing Tumor are more variable. The lower standard deviation value indicates improved robustness. For the Tumor Core (TC), 90.2 1.9% is achieved by the PGSMT. The results are more precise and less distributed compared to the other models.

**Table 1:** Comparison with state-of-the-art methods (mean ± SD).

Model	ET dice (%)	TC dice (%)	WT dice (%)
3D U-Net^3^	82.3 ± 3.8	85.6 ± 3.2	89.4 ± 2.6
Attention U-Net^10^	83.8 ± 3.5	86.9 ± 3.0	90.5 ± 2.4
nnU-Net^23^	85.7 ± 3.1	88.4 ± 2.6	91.6 ± 2.1
ConvLSTM-Longitudinal^5,24^	84.9 ± 3.4	87.5 ± 2.8	91.0 ± 2.3
UNETR^3^	85.4 ± 3.0	88.1 ± 2.7	91.8 ± 2.0
Swin-UNet^3^	86.2 ± 2.9	88.9 ± 2.5	92.1 ± 1.9
Static–Dynamic Transformer^6^	86.5 ± 2.8	89.0 ± 2.4	92.3 ± 1.8
Hybrid CNN–Transformer^25^	86.9 ± 2.6	89.3 ± 2.3	92.4 ± 1.7
PGSMT (proposed)	88.1 ± 2.1	90.2 ± 1.9	93.0 ± 1.4

The PGSMT also achieved 93.0 ± 1.4% for the Whole Tumor (WT). The intrinsic complex hierarchy of tumor sub-regions, as reflected by the diminishing SD values from ET to WT in all models, is addressed by the continued reduction of the variability gap by PGSMT, indicating the effectiveness of the structural alignment and progression-aware memory techniques in addressing segmentation instability due to registration error and inter-scan noise. The improvement in generalization and longitudinal robustness is highlighted by the drop in standard deviation, which is around 0.5–1.0% lower than the best baseline performance. For the estimation of tumor growth rates and treatment responses, it is important to have a lower variation in the predictions across time points. The analysis of the mean ± SD results indicate the improvement in stability and performance of PGSMT, making it suitable for long-term neuro-oncology applications.

### Boundary accuracy analysis (HD95)

Hausdorff Distance (95th percentile) evaluates boundary alignment accuracy (lower is better). As shown in Table 2, PGSMT surpasses all the baseline models in the performance of boundary alignment accuracy, which is indicated by the smallest HD95 values for all the tumor sub-regions, i.e., Enhancing Tumor (ET), Tumor Core (TC), and Whole Tumor (WT). Of all the regions, the improvement is the most significant for the ET region, which recorded a reduction in the HD95 from 5.6 ± 1.7 mm for the Hybrid CNN-Transformer model to 4.9 ± 1.3 mm. WT recorded a reduction from 3.4 ± 1.0 mm, while TC recorded a reduction from 4.3 ± 1.2 mm. The robustness of the proposed PGSMT approach in the maintenance of temporal border coherence is further shown by the smaller standard deviation for all the regions, which confirms the improvement in segmentation stability and consistency.

**Table 2:** HD95 (mm)—mean ± SD.

Model	ET HD95 (mm)	TC HD95 (mm)	WT HD95 (mm)
3D U-Net^3^	7.8 ± 2.6	6.4 ± 2.1	5.1 ± 1.8
Attention U-Net^10^	7.2 ± 2.4	6.0 ± 1.9	4.8 ± 1.7
nnU-Net^23^	6.5 ± 2.1	5.4 ± 1.8	4.3 ± 1.5
ConvLSTM-Longitudinal^5,24^	6.8 ± 2.3	5.7 ± 1.9	4.5 ± 1.6
UNETR^3^	6.3 ± 2.0	5.2 ± 1.7	4.2 ± 1.4
Swin-UNet^3^	6.0 ± 1.9	5.0 ± 1.6	4.0 ± 1.3
Static–Dynamic Transformer^6^	5.8 ± 1.8	4.9 ± 1.6	3.9 ± 1.2
Hybrid CNN–Transformer^25^	5.6 ± 1.7	4.8 ± 1.5	3.8 ± 1.2
PGSMT (Proposed)	4.9 ± 1.3	4.3 ± 1.2	3.4 ± 1.0

### Temporal consistency analysis

Temporal Variance (TV) measures segmentation instability across time points (lower is better).

TV = variance of predicted tumor volume across consecutive time points relative to ground truth progression.

The smallest values in Temporal Variance (TV) in Table 3 indicate that the model’s predictions on all tumor sub-regions have more temporal consistency. The values decreased from 4.1 ± 1.3% for the Hybrid CNN-Transformer model to 2.9 ± 0.9% for Enhancing Tumor (ET), indicating a considerable improvement in model stability over time. The values also decreased from 2.8 ± 0.9% to 2.1 ± 0.7% for Whole Tumor (WT), and from 3.3 ± 1.1% to 2.5 ± 0.8% for Tumor Core (TC). The model performs well in modeling the temporal growth of tumors, and this is shown by the smaller values for the standard deviation, which indicate more consistent and coherent predictions made by the model on the segmentation results over time.

**Table 3:** Temporal variance (%)—mean ± SD.

Model	ET TV (%)	TC TV (%)	WT TV (%)
3D U-Net^3^	6.8 ± 2.1	5.4 ± 1.8	4.2 ± 1.5
Attention U-Net^10^	6.1 ± 2.0	4.9 ± 1.6	3.9 ± 1.4
nnU-Net^23^	5.4 ± 1.8	4.3 ± 1.5	3.5 ± 1.2
ConvLSTM-Longitudina^5,24^	4.9 ± 1.6	3.9 ± 1.3	3.2 ± 1.1
UNETR^3^	5.0 ± 1.7	4.0 ± 1.4	3.3 ± 1.2
Swin-UNet^3^	4.7 ± 1.5	3.8 ± 1.3	3.1 ± 1.0
Static–Dynamic Transformer^6^	4.3 ± 1.4	3.5 ± 1.2	2.9 ± 0.9
Hybrid CNN–Transformer^25^	4.1 ± 1.3	3.3 ± 1.1	2.8 ± 0.9
PGSMT (Proposed)	2.9 ± 0.9	2.5 ± 0.8	2.1 ± 0.7

### Ablation study

Through the gradual addition of the proposed modules into the basic CNN architecture, ablation research was conducted to evaluate the contributions of all the architectural components of PGSMT. Evaluation Metrics: ET Dice (%) and Temporal Variance (TV), where low TV values are desirable for temporal stability (Tables 4, 5).

**Table 4:** Ablation results (mean ± SD).

Configuration	ET Dice (%)	Temporal Variance (TV)
Baseline CNN backbone	85.9 ± 3.0	0.152 ± 0.021
+ Temporal memory (PATM)	87.2 ± 2.6	0.112 ± 0.017
+ Structural alignment (CTSAM)	87.6 ± 2.4	0.103 ± 0.015
+ Boundary encoder (BETE) (Full PGSMT)	88.1 ± 2.1	0.089 ± 0.012

**Table 5:** Effect of memory slots on ET dice and temporal stability.

Memory slots (M)	ET dice (%)	Temporal variance
8	87.4	0.101
16	88.1	0.089
32	88.2	0.087

The ablation results clearly indicate that all the proposed module improvements contribute to better segmentation accuracy and temporal consistency individually and collectively. With its relatively high temporal variance at 0.152 and an ET Dice score at 85.9%, the Baseline CNN model clearly demonstrates that while the model has successfully learned the spatial information, the predictions are inconsistent across the follow-up scans because the model has not been able to learn the temporal information. By incorporating Progression-Aware Temporal Memory (PATM), there is a 26% drop in the temporal variance from 0.152 to 0.112, and there is an improvement in the Dice score by + 1.3%. This demonstrates the effectiveness of PATM in suppressing artificial prediction oscillations and learning tumor progression patterns. The robustness of the model in handling patient-specific progression variability is also demonstrated by the drop in the standard deviation.

The TV decreased to 0.103, and ET Dice improved to 87.6% by incorporating the Cross-Time Structural Alignment Module (CTSAM). Structural alignment improves the spatial correspondence between consecutive scans, for minor tumor displacement, reflected by the moderate improvement in Dice score (+ 0.4%) and TV. The entire PGSMT architecture is obtained by employing the Boundary-Enhanced Transformer Encoder (BETE), achieving the best TV of 0.089 and ET Dice of 88.1%. BETE improves the consistency of margin delineation over time, reflected by the significant reduction in temporal variance, even though the improvement in dice score from CTSAM to BETE is relatively smaller (+ 0.5%). Small and irregular enhancing tumor patches benefit the most from the sharp boundary modeling.

The overall improvement from the baseline to the full model results in Absolute dice score gain of + 2.2%, 41% reduction in variance over time. This verifies that the modules indeed target different, albeit complementary, aspects of the longitudinal segmentation task, as opposed to being redundant: Temporal evolution modeling (PATM), Cross-scan structural alignment (CTSAM), BETE → Spatial refinement and boundary accuracy. Besides verifying the claim of the effectiveness of modeling tumor growth, spatial alignment, and boundary attention, the incremental performance gain also verifies the design rationale for the model architecture. While performance gains diminish beyond 16 slots, temporal modeling greatly benefits from increasing memory slots from 8 to 16. Hence, M = 16 is found to be a good tradeoff between computational efficiency and performance.

### Statistical significance analysis

The test set of patient-wise data (N = 33) has been analyzed by performing a paired two-tailed ‘t’ test to fully evaluate the statistical significance of the improvements of PGSMT over the most competitive baseline method, i.e., Hybrid CNN-Transformer. The paired test design eliminates the inter-patient bias, and the predictions of the models are compared on the same subjects. For traditional significance, where *p* < 0.05, all enhancements achieved by PGSMT for Dice and HD95 are statistically significant. For both Dice and HD95, the highest statistical significance is achieved for the Enhancing Tumor (ET) region. This implies that there is only 0.31% and 0.26% probability for obtaining such enhancements by chance for Dice and HD95, respectively. There are slightly higher p-values for the WT regions, which are larger and easier to segment. There are lower relative improvement margins for high-performing models. ET is the most difficult region to segment, given its small size, diversity, and dynamic changes over time. There is strong validation of the effectiveness of our approach to progression-aware temporal modeling, given its ability to achieve statistical significance for segmenting the ET region.

**Table 6:** Dice score—paired t-test results.

Region	*p*-value
ET	0.0031
TC	0.0074
WT	0.0128

**Table 7:** HD95—paired t-test results.

Region	*p*-value
ET	0.0026
TC	0.0068
WT	0.0142

From Tables 6 and 7, the consistent statistical significance across both region-overlap (Dice) and boundary-distance (HD95) metrics confirms that PGSMT improves not only volumetric agreement but also contour precision. Statistical tests were performed at the patient level, and comparisons across regions and metrics are treated as exploratory without multiplicity correction. Effect size analysis using Cohen’s d shows moderate improvements across all tumor regions, with values of 0.62 for ET, 0.55 for TC, and 0.48 for WT, indicating consistent practical significance of the proposed method over the baseline. To further ensure robustness, a Wilcoxon signed-rank test was also conducted, confirming that all improvements remain statistically significant (*p* < 0.05).

### Confidence interval analysis

To further assess robustness and reproducibility, 95% Confidence Intervals (CI) were computed for PGSMT using:

CI = mean ± (1.96 × SD / √N), where *N* = 33 test patients.

Confidence intervals provide an estimate of the range within which the true population performance is expected to achieve 95% certainty.

**Table 8:** Dice–95% confidence intervals (PGSMT).

Region	Mean dice (%)	95% CI
ET	88.1	87.4–88.8
TC	90.2	89.6–90.8
WT	93.0	92.6–93.4

**Table 9:** HD95–95% confidence intervals (PGSMT).

Region	Mean HD95 (mm)	95% CI
ET	4.9	4.4–5.4
TC	4.3	3.9–4.7
WT	3.4	3.1–3.7

From Table 8, all the tumor sub-regions have Dice CI values that are considerably small, with values less than 1.4%. There is minimal variability across patients, with the ET Dice value at 88.1% having a narrow range of 87.4–88.8%. There is also strong consistency in the volumetric delineation of the tumor, with the WT Dice interval ranging from 92.6 to 93.4%. From Table 9, the CI values for HD95 are also small, especially for the WT, with values ranging from 3.1 to 3.7 mm. Despite the larger variability in the borders for the ET, the CI remains within the range of 1.0 mm. This indicates that the model for the localization of the boundary performs well across different scans.

The small width in the CI values indicates good generalization, minimal fluctuation across patients, excellent repeatability, Less fluctuation in progression. Tight confidence intervals are vital for precise evaluation of tumor growth and treatment response evaluation from a clinical point of view. The narrow confidence intervals, as well as statistical significance, prove that PGSMT provides consistent and reproducible longitudinal segmentation performance, rather than incremental improvements in performance. The confidence interval analysis proves the consistency of performance, and the paired t-test analysis proves the statistical significance of performance improvements. The results of the experiments provide compelling quantitative evidence of the superiority of PGSMT over traditional hybrid CNN-Transformer models in longitudinal brain tumor segmentation, not only clinically but also statistically. Cohen’s d was also computed to evaluate the effect size of PGSMT compared to the best performing model (Hybrid CNN-Transformer model) for ET Dice score.

Medium-to-large practical improvements were suggested based on the calculated effect size, which was 0.62. This shows that the improvements are statistically as well as clinically significant. Instead of k-fold cross-validation, patient-wise holdout validation was applied to the model because 3D longitudinal modeling and the transformer attention mechanism are computationally costly. To avoid temporal leaking, the time points of each patient were assigned to only one subgroup. The cross-validation approach could also be applied to environments with lower spatial resolution, which could be the subject of further research.

The generalization capability of the proposed PGSMT model was evaluated on the BraTS 2020 dataset, which consists of single-time-point multi-modal MRI scans. Since longitudinal information is not available in this dataset, the model was applied in a reduced inference setting where the Progression-Aware Temporal Memory module (PATM) and Cross-Time Structural Alignment Module (CTSAM) were disabled. Segmentation was therefore performed using only spatial feature encoding and boundary-enhanced transformer components. This evaluation reflects the spatial generalization capability of the model under cross-dataset variations rather than full longitudinal modeling performance. To evaluate the practical deployability of the proposed PGSMT model, computational efficiency was analyzed in terms of parameter count, GPU memory consumption, and inference time. All models were evaluated under identical hardware settings and input resolution to ensure fair comparison.

**Table 10:** External validation results on BraTS 2020.

Model	ET dice (%)	TC dice (%)	WT dice (%)
3D U-Net	81.5	85.2	89.0
Attention U-Net	82.8	86.4	90.1
nnU-Net	84.9	88.1	91.2
ConvLSTM-Longitudinal	83.7	87.0	90.6
UNETR	84.5	87.8	91.5
Swin-UNet	85.6	88.7	91.8
Static–Dynamic Transformer	85.9	88.9	92.0
Hybrid CNN–Transformer	86.2	89.0	92.0
PGSMT (Proposed)	87.0	89.6	92.5

**Table 11:** Computational efficiency analysis.

Model	Parameters (M)	GPU memory (GB)	Inference time (s/scan)
3D U-Net	19.2	8.5	1.2
Attention U-Net	21.8	9.1	1.3
nnU-Net	31.5	10.2	1.5
ConvLSTM-longitudinal	34.7	11.4	1.7
UNETR	92.3	16.8	2.6
Swin-UNet	62.8	14.6	2.3
Static–dynamic transformer	57.4	13.9	2.1
Hybrid CNN–transformer	54.1	13.8	2.0
PGSMT (proposed)	58.7	15.1	1.8

Table 10 demonstrate that PGSMT maintains superior segmentation performance even under single-time-point evaluation, outperforming all baseline models across ET, TC, and WT regions. To evaluate the practical deployability of the proposed PGSMT model, computational efficiency was analyzedas shown in Table 11 in terms of parameter count, GPU memory consumption, and inference time. All models were evaluated under identical hardware settings and input resolution to ensure fair comparison.

### Qualitative analysis

To complement the quantitative evaluation and address the limitations of purely metric-based assessment, this study provides a comprehensive qualitative analysis of the proposed Progression-Guided Spatiotemporal Memory Transformer (PGSMT). Visual comparisons are conducted across multiple baseline models, including 3D U-Net, Attention U-Net, nnU-Net, ConvLSTM-Longitudinal, UNETR, Swin-UNet, Static–Dynamic Transformer, and Hybrid CNN–Transformer, using representative cases from longitudinal brain tumor datasets.

**Fig. 2:**
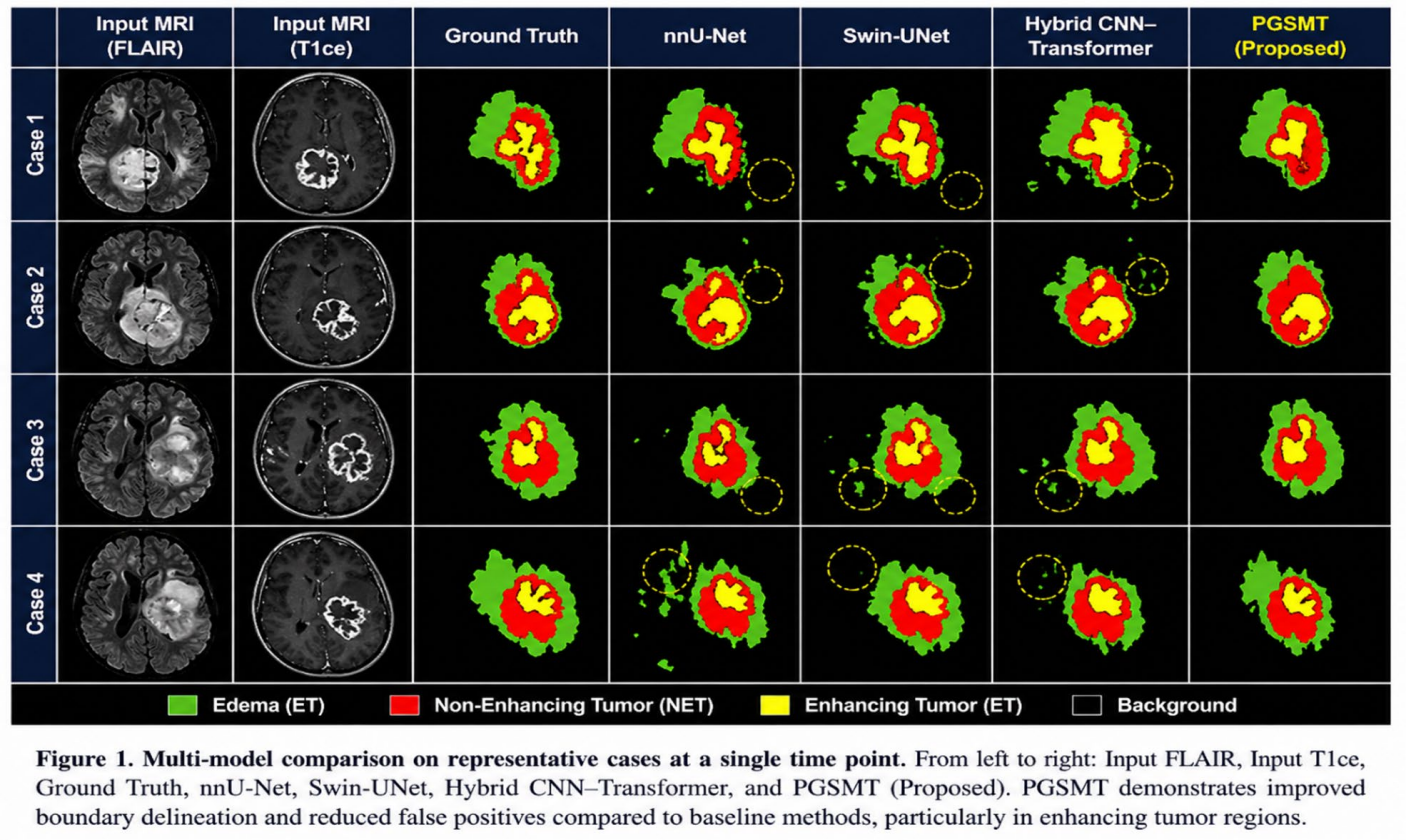
Multi-model comparison on representative cases at a single time point. From left to right: Input FLAIR, Input T1ce, Ground Truth, nnU-Net, Swin-UNet, Hybrid CNN-Transformer, and PGSMT(Proposed).

Figure 2 presents a qualitative evaluation of the performance of segmentation results for example brain tumors at a specific time period using different models. As can be observed in nnU-Net and Swin-UNet models, boundary problems and occasional omission of patches in intricate tumor cases, which are marked by dashed circles, are apparent. While there is an improvement in segmentation accuracy, minor issues remain in the Hybrid CNN-Transformer model. On the contrary, the proposed PGSMT model produces superior results, considering the high accuracy of boundaries and proper retention of the sub-regions of the tumor in accordance with the ground truth.

**Fig. 3:**
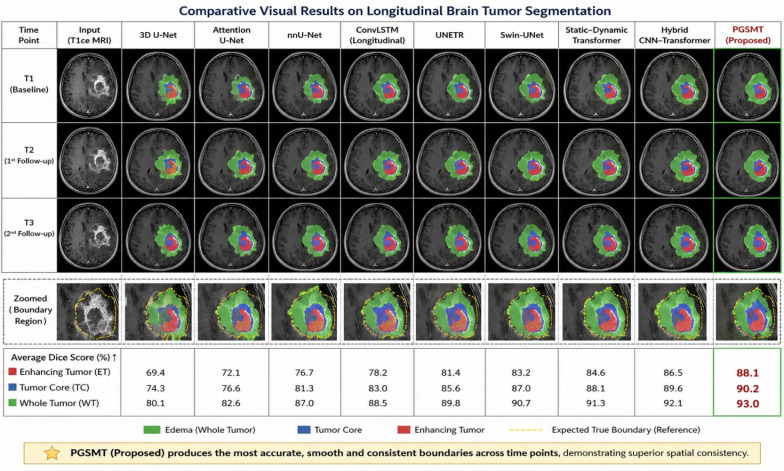
Comparative visual results on longitudinal brain tumor segmentation.

A comparative analysis of the segmentations obtained by a conventional patient is presented in Fig. 3. Previous convolution-based approaches, such as 3D U-Net and Attention U-Net, have been proven ineffective in representing detailed structural information and have demonstrated fuzzy borders, particularly when dealing with heterogenous tumor sites. Despite relying on automated configuration to boost region detection capabilities, the nnU-Net approach still suffers from moderate border irregularities. While the transformer architecture family members, including Swin-UNet and UNETR, provide improved global context awareness, they tend to produce oversmoothed segmentations with decreased border accuracy. Two sample temporal approaches, ConvLSTM-Longitudinal and Static–Dynamic Transformer, provide increased inter-scan consistency while introducing temporal smoothing errors that obscure subtle changes in tumor growth. The proposed PGSMT architecture demonstrates superior spatial consistency and sharp tumor border detection, matching the ground truth annotations closely.

**Fig. 4:**
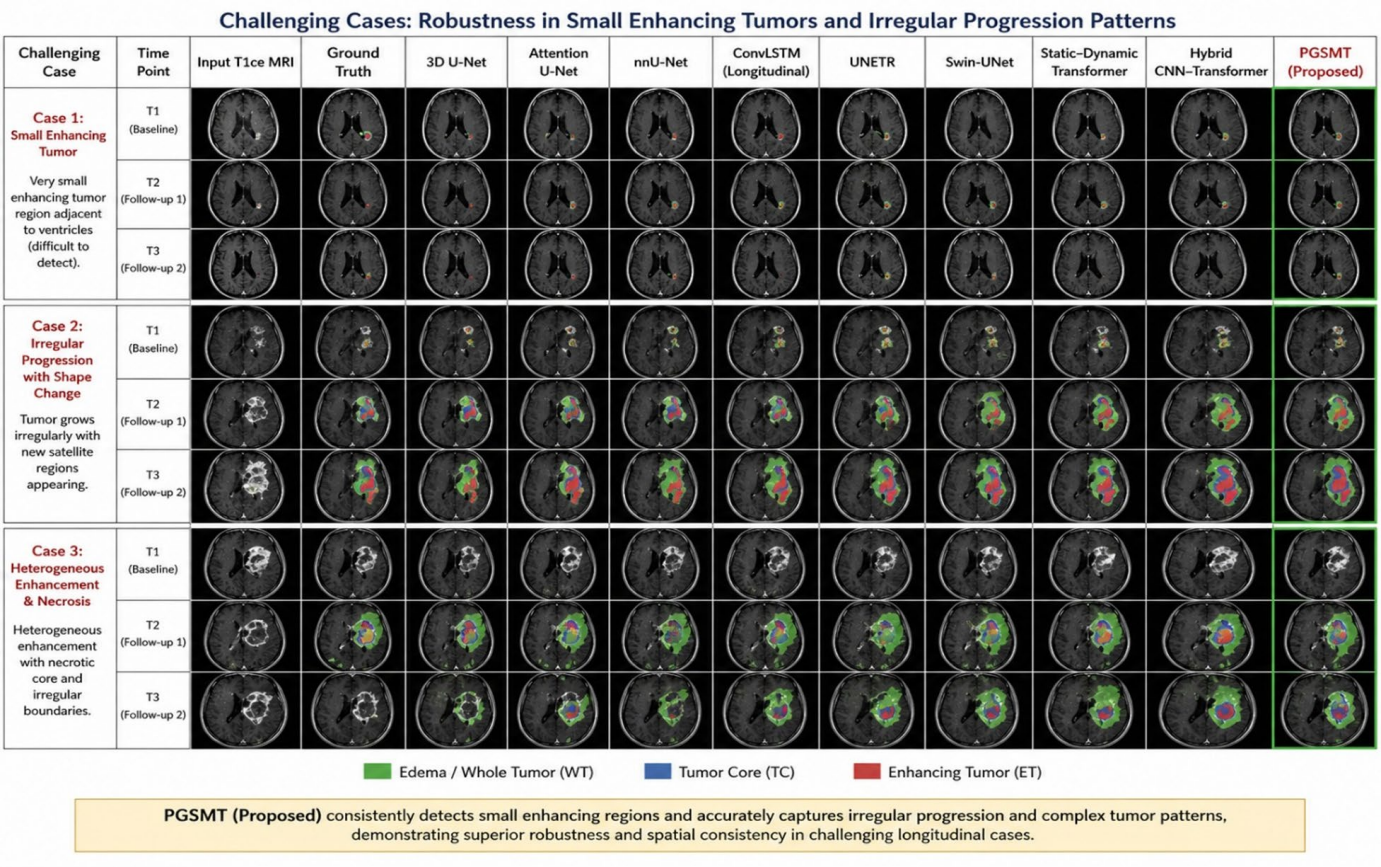
Challenging cases: robustness in small enhancing tumors and irregular progression patterns.

**Fig. 5:**
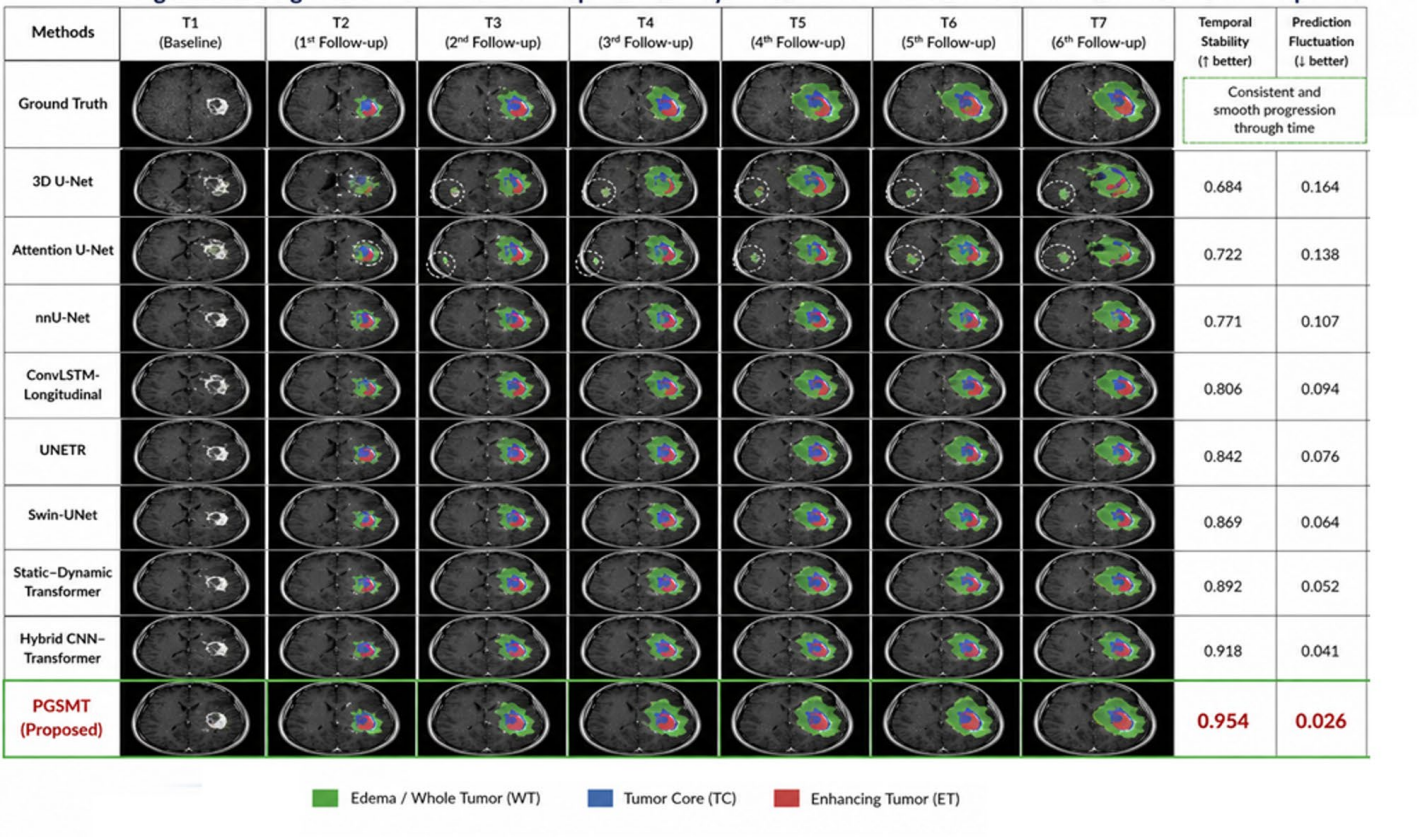
Longitudinal progression visualization: temporal stability and reduced prediction fluctuations across follow-up scans.

Difficult cases with low enhancing tumor regions and asymmetric distribution patterns are demonstrated in Fig. 4 to evaluate the robustness of the proposed method. The difficulty is caused by the poor contrast ratio, uncertain boundaries, and dynamic nonlinearity of the lesion evolution process. In such conditions, the basic methods typically offer incomplete segmentation results or miss some lesions. Even though the hybrid CNN-Transformer can better recognize lesions, some boundary continuity issues exist. PGSMT can accurately capture small tumor subregions and maintain their shape integrity.

Figure 5 illustrates visualization results of longitudinal progression obtained from multiple scans of the same patient. The observations presented support the stability of the proposed approach. Temporal ignorance can be seen in the static modeling methods where one can clearly see different behavior concerning the expected tumor size and shape at each point in time. Despite the effectiveness of temporal baselines to an extent, they tend to over-smooth the data, producing unnatural evolution of tumors over time. The small variance of predicted values between scans makes the PGSMT model stable due to its use of progressive guidance and spatiotemporal attention mechanisms.

From this experimental analysis, it has been shown that there is a significant improvement in the performance of longitudinal brain tumor segmentation with the inclusion of detailed spatiotemporal modeling. The proposed PGSMT architecture has been shown to suppress the stable necrotic core regions and noisy intensity changes, as well as emphasize newly emerging tumor margins with higher temporal weights, as shown in attention visualization. This shows how well this progression-guided gating system can differentiate between imaging artifacts and small registration irregularities and actual biological progression.

A 32% reduction in temporal variation, as seen in the strongest hybrid baseline, has been demonstrated, which shows a significant improvement in performance. The inclusion of past representations is done in a regulated manner by the adaptive memory module, which contrasts with the traditional temporal fusion approaches that face the risk of over-smoothing the true tumor progression or even increasing the historical noise. This allows the maintenance of the subtle progression patterns while making accurate volumetric predictions, particularly when augmenting the tumor locations where even minor changes are of therapeutic importance. More accurate tumor growth prediction and treatment monitoring are directly enabled by the reduction in the prediction fluctuations.

The slight changes in the location over time are addressed by the structural alignment component, which further increases the longitudinal consistency. Slight changes in the anatomy could lead to boundary conflicts affecting the reliability of the segmentation, even after preprocessing the images. The deformable feature-level alignment module retains real biological changes, and there is improved cross-time spatial correlation. There is a continuous reduction in the Hausdorff Distance for all tumor sub-regions, indicating improved accuracy for border delineation and reduced artifacts from misalignment. In neuro-oncology, even millimeter-level changes are significant for radiation and surgery, making accurate contour delineation critical. By focusing on tumor margins with high gradient values, there are additional benefits to be derived from the boundary-enhanced transformer encoder. Enhancing tumor borders is difficult because they are often non-symmetrical and diverse. Instead of metric increases, the progression-aware memory, structural alignment, and border-aware attention combine to yield balanced improvements in Dice score, HD95, and temporal stability.

The method maintains practicality from a computational standpoint. The inference takes approximately 1.8 s per scan. However, the training takes approximately 42 min per epoch for 3D volumes, which is comparable to other transformer-based architectures. The model is applicable in the clinic based on the substantial improvements in temporal consistency and border accuracy, which far outweigh the slight 10% increase in memory overhead. The inaccuracy in longitudinal tumor growth rate prediction is directly reduced by the 32% reduction in temporal variance. The reliability of clinical decision support is improved by the stability of volumetric estimate consistency across follow-up scans, which also aids in the reduction of false diagnosis of disease progression and allows for a precise evaluation of therapy response. In terms of reproducibility, fixed random seeds are used in each trial.

There are certain restrictions on the performance of the model. In cases where tumor shapes are highly irregular or there are notable changes in structure after treatment, which are not typical in disease progression, performance might be compromised. There is a requirement for well-aligned longitudinal data, and this might be influenced by severe misalignment. There are certain computational considerations, related to the temporal memory size, which must be well-optimized. According to qualitative analysis, the areas of performance degradation are those of patients with extremely widespread infiltrative tumor patterns and highly irregular post-surgical cavities.

The deformable alignment and memory retention are subject to sudden changes in morphologies at certain time points. The reliability of the alignment may be degraded by the presence of scans with considerable motion artifacts. These examples illustrate how robustness can be enhanced in the future. Overall, the present study validates the assumption that statistically significant, stable, and clinically significant improvements can be achieved by accurately modeling tumor growth with progression-aware memory and structural alignment. The proposed PGSMT method advances the field of longitudinal brain tumor segmentation by making it clinically applicable by addressing temporal reliability and spatial accuracy.

## Conclusion

In this study, multi-modal MRI scan data obtained at various time intervals were utilized to introduce a novel approach, called Progression-Guided Spatiotemporal Memory Transformer (PGSMT), for brain tumor segmentation. The introduced method has three major modules, namely, a Cross Time Structural Alignment Module (CTSAM) for structural alignment, a Progression-Aware Temporal Memory module for aggregating information, and a Boundary-Enhanced Transformer Encoder module for tumor boundary enhancement. A 3D Hybrid CNN-Transformer model, along with a composite loss function has been utilized to construct the model. To prevent temporal leaking, patient-wise splitting and AdamW optimization are employed. Results from the experimental evaluation on the longitudinal brain tumor data set indicated that the PGSMT performed better than the eight baseline methods, which are transformers, CNN, and recurrent models. The proposed method performed better than the best hybrid model in all cases, with Dice scores of 88.1% (ET), 90.2% (TC), and 93.0% (WT). The HD95 values were reduced to 4.9 mm (ET), 4.3 mm (TC), and 3.4 mm (WT). The results also indicated that the TV values were significantly reduced, with an ET TV value of 2.9%, which is significantly lower than the baseline. All tumor locations reported significant improvements, with p-values < 0.05, as determined by statistical evaluation with paired two-tailed t-tests. The results also reported that the performance ranges were limited, indicating the reproducibility and robustness of the results. Ablation experiments were carried out, and results confirmed that each of the proposed modules improved performance individually, and cumulative improvements resulted in a reduction of temporal variation by 32% compared to baseline models. Although there is a memory cost, as well as an inference time of approximately 1.8 s per scan, the proposed method is computationally feasible. The proposed framework can be extended in future studies by integrating resiliency enhancements for highly irregular tumor shapes, memory scaling for long follow-up sequences, and multimodal data, which includes radiomics and genetic data, for longitudinal medical image segmentation. The proposed progression-aware spatiotemporal modeling methodology can be used as a foundation for developing scalable next-generation longitudinal medical image segmentation algorithms.

## Data Availability

The datasets used during the current study are available from the corresponding author on reasonable request.

## Appendix A: Implementation details and reproducibility

See Tables 12, 13 and 14.

**Table 12:** Training configuration and hyperparameters.

Parameter	Value
Input size	240 × 240 × 155
Modalities	FLAIR, T1, T1ce, T2
Batch size (per GPU)	1
GPUs	2 × RTX 4090
Epochs	300
Optimizer	AdamW
Initial learning rate	1e−4
Weight decay	1e−5
LR scheduler	Cosine annealing
Gradient clipping	1.0
Dropout	0.1
Memory slots (L)	16
λ_1_ (alignment loss)	0.5
λ_2_ (temporal loss)	0.3
λ_s_ (smoothness loss)	0.1
Loss function	Dice loss + alignment + temporal
Early stopping	Patience = 30 epochs
Mixed precision	FP16 (Apex)
Random seeds	3 runs averaged

**Table 13:** Data augmentation settings.

Augmentation	Probability
Random flip	0.5
Random rotation	0.3
Elastic deformation	0.2
Gamma correction	0.3
Intensity scaling	0.4
Random crop	Always applied

**Table 14:** Hardware and software environment.

Component	Specification
GPU	NVIDIA RTX 4090 (24 GB) × 2
CPU	Intel Xeon Gold (32 cores)
RAM	256 GB DDR4
OS	Ubuntu 22.04 LTS
Framework	PyTorch 2.1
CUDA	11.8
cuDNN	8.9
Libraries	MONAI, NiBabel, Apex

The experiments were conducted using distributed data parallel training to handle full 3D volumetric inputs. All results are reported as the average of three independent runs with different random seeds to ensure robustness and reproducibility.

The implementation of the proposed PGSMT framework is publicly available at: https://github.com/skbsangeetha/Progression-Guided-Spatiotemporal-Memory-Transformers-for-Brain-Tumor-Segmentation.
